# First Description of the Hyperpnea–Hypopnea Periodic Breathing in Patients with Interstitial Lung Disease-Obstructive Sleep Apnea: Treatment Implications in a Real-Life Setting

**DOI:** 10.3390/ijerph16234712

**Published:** 2019-11-26

**Authors:** Angelo Canora, Carmine Nicoletta, Giacomo Ghinassi, Dario Bruzzese, Gaetano Rea, Annalisa Capaccio, Sabrina Castaldo, Antonietta Coppola, Giorgio Emanuele Polistina, Alessandro Sanduzzi, Marialuisa Bocchino

**Affiliations:** 1 Dipartimento di Medicina Clinica e Chirurgia, Sezione di Malattie dell’Apparato Respiratorio, Università Federico II, 80131 Napoli, Italy; a.canora@hotmail.it (A.C.); carminenicoletta1983@gmail.com (C.N.); giacomo.ghinassi87@gmail.com (G.G.); annalisacapaccio@libero.it (A.C.); sabry.castaldo@gmail.com (S.C.); antonietta.coppola84@gmail.com (A.C.); giorgiopolistina@gmail.com (G.E.P.); sanduzzi@unina.it (A.S.); 2 Dipartimento di Sanità Pubblica, Università Federico II, 80131 Napoli, Italy; dbruzzes@unina.it; 3 Dipartimento dei Servizi Diagnostici e Generali, Ospedali dei Colli, Monaldi-Cotugno, 80131 Napoli, Italy; g.rea71@gmail.com

**Keywords:** obstructive sleep apnea, idiopathic pulmonary fibrosis, interstitial lung disease, hypopnea, hyperpnea, oxygen

## Abstract

There is evidence that hypopneas are more common than apneas in obstructive sleep apnea (OSA) related to idiopathic pulmonary fibrosis (IPF). We investigated the frequency distribution of hypopneas in 100 patients with interstitial lung diseases (ILDs) (mean age 69 yrs ± 7.8; 70% males), including 54 IPF cases, screened for OSA by home sleep testing. Fifty age- and sex-matched pure OSA patients were included as controls. In ILD-OSA patients the sleep breathing pattern was characterized by a high prevalence of hypopneas that were preceded by hyperpnea events configuring a sort of periodic pattern. This finding, we arbitrarily defined hyperpnea–hypopnea periodic breathing (HHPB), was likely reflecting a central event and was completely absent in control OSA. Also, the HHPB was highly responsive to oxygen but not to the continuous positive pressure support. Thirty-three ILD-OSA patients (42%) with a HHPB associated with a hypopnea/apnea ratio ≥3 had the best response to oxygen with a median residual AHI of 2.6 (1.8–5.6) vs. 28.3 (20.7–37.8) at baseline (*p* < 0.0001). ILD-OSA patients with these characteristics were similarly distributed in IPF (54.5%) and no-IPF cases (45.5%), the most of them being affected by moderate–severe OSA (*p* = 0.027). Future studies addressing the pathogenesis and therapy management of the HHPB should be encouraged in ILD-OSA patients.

## 1. Introduction

Interstitial lung diseases (ILDs) include more than 200 disorders with considerable variation of clinical course, treatment, and prognosis. Approximately two-thirds do not have a known cause, the others resulting from environmental/occupational exposure, infections, drugs, and radiation [1]. Despite recent advances in pharmacotherapy [2], many ILDs have limited treatment options. Among the idiopathic interstitial pneumonia, idiopathic pulmonary fibrosis (IPF) is the most common form affecting 30 persons per 100,000 people in the general population, and as many as 175 per 100,000 people in the age group of >75 years [3]. IPF is a poor prognosis disease with a progressive and debilitating clinical behavior. Co-morbidities, ranging from gastro-esophageal reflux to lung cancer, are frequently associated with IPF with a significant impact on disease outcome [4,5]. Recent observations suggest a close association between sleep-related respiratory disorders and ILDs [6]. The combination of obstructive sleep apnea (OSA) and IPF has received much attention as representing a significant cause of quality of life deterioration and mortality [7,8,9,10,11,12,13,14]. Despite progress in raising awareness of OSA, it remains under-diagnosed in the general population with prevalence estimates of 22% in men and 17% in women [15]. Episodes of intermittent hypoxia and sleep fragmentation occur during nocturnal cycles of pharyngeal collapse, leading to a cascade of maladaptive processes, like systemic inflammation, endothelial dysfunction, and oxidative stress [16,17,18]. Alterations in sleep macro- and micro-architecture have been observed in IPF patients [7,10,14,19], with OSA being more common among cases with a higher body mass index (BMI) [4]. Interestingly, Lancaster et al. found in 2009 that hypopneas were more common than apneas in 44 IPF patients affected by OSA [8]. Hypopneas, as apneas, were balanced during REM and non-REM sleep. A high frequency of hypopneas had also previously been reported in a small cohort of 37 ILD patients in a Turkish study [20].

As no efforts that we are aware of have further described the prevalence of hypopneas in this clinical scenario, our purpose was to investigate the frequency and type distribution of hypopnea events in a cohort of ILD patients, including IPF, screened for OSA by home sleep testing in a real-life setting. Therapy implication was also evaluated.

## 2. Materials and Methods

### 2.1. Study Population

The study population was composed of 100 consecutive patients with clinically stable fibrotic ILDs referring to our Division at the time of first diagnosis. They included 54 treatment naïve IPF patients, according to the 2011 official diagnostic criteria [21], and 46 patients suffering from other untreated ILDs, including idiopathic non-specific interstitial pneumonia (n = 19), connective tissue disease-related ILDs (n = 9), chronic hypersensitivity pneumonia (n = 12), and smoking-related ILDs (n = 6). Fifty age- and sex-matched OSA patients with no concomitant lung diseases were included as control group. The study was conducted in accordance with the amended Declaration of Helsinki after approval by the local institutional ethics committee (Protocol n. 1129, August 4th 2015). Patients gave their written informed consent and all data were collected in an anonymous way. Spirometry, lung volumes measurement, and determination of the hemoglobin (Hb)-adjusted single-breath diffusing lung capacity of the carbon monoxide (DLCOsb) were performed using a computer-assisted spirometer (Quark PFT 2008 Suite Version Cosmed Ltd., Rome, Italy) according to international standards [22,23,24]. The 6-min walk test (6-MWT) was performed by trained hospital staff according to guidelines [25]. Systolic pulmonary arterial pressure (sPAP) was measured by conventional trans-thoracic echocardiography (Philips iE33 ultrasound machine, Philips Medical Systems, Andover, MA, USA) [26] with estimation of pulmonary hypertension according to international guidelines [27].

### 2.2. OSA Assessment

Sleep related respiratory events were recorded by home sleep testing with a VitalNight data acquisition and analysis system (AirLiquide Medical System, Rangendigen, Germany). Measurements included chest wall and abdominal movements, nasal airflow, oxygen saturation (SpO_2_), heart rate, and body position. Respiratory data included the number of the events, i.e., obstructive and central apneas, mixed apneas, obstructive and central hypopneas, the apnea-hypopnea index (AHI), the central apnea index (CAI), the obstructive apnea index (OAI), the hypopnea index (HI), the oxygen desaturation index (ODI), and the percentage of time spent with SpO_2_ < 90% (t90%) [28]. The total sleep time (TST) and the percentage of time in supine position were recorded as well. Home sleep testing was realized while patients were breathing ambient air with patients with severe hypoxemia at rest (arterial pO_2_ < 55 mmHg) being excluded. Sleep reports were analyzed by two physicians with more than 3-years’ experience in the field to exclude any artifact [28]. Before testing, all participants were also asked to complete the Epworth sleepiness scale (ESS) questionnaire. An ESS score >10 was considered to be consistent with an excessive daytime sleepiness [29]. Technical definitions of sleep related respiratory events: (1) obstructive apnea when airflow drops by more than 90% from baseline for at least 90% of the entire respiratory event with preserved chest and/or abdominal movements, for the duration of at least 10 s; (2) central apnea when airflow completely stops in the absence of any respiratory and abdominal effort for a minimum of 10 s; (3) mixed apnea when airflow drops by more than 90% from baseline for at least 90% of the entire respiratory event, for a minimum duration of 10 s, associated with no inspiratory effort in the initial portion of the event, and followed by resumption of inspiratory effort before the end of the event; (4) hypopnea when airflow drops at least 30% from baseline for a duration of at least 10 s, accompanied by a minimum 4% drop in SpO_2_; (5) obstructive hypopnea when the event is associated to snoring or increased inspiratory flattening, or thoraco-abdominal paradox; (6) central hypopnea when snoring, increased inspiratory flattening, and thoraco-abdominal paradox are absent; (7) t90% is the time spent with a SpO_2_ <90%, expressed as percentage of the entire sleep time. OSA was defined as an AHI >5 per hour and disease severity was graded according to accepted criteria [28]. Nocturnal respiratory failure (NRF) was defined if t90% was ≥30% or there was at least one period of 5 min minimum with a SpO_2_ ≤90% with a nadir of 85% during registration [28]. Titration of continuous positive airway pressure (CPAP) was performed in patients affected by moderate and severe OSA for three consecutive nights with auto-CPAP disposal (Dreamstar Info, Sefam, Nancy, France) [28]. Sleep oxygen supplementation was administered in selected cases, as described below.

### 2.3. Statistical Analysis

Numerical variables were described using the mean ± standard deviation (SD) in case of symmetrical distribution or the median with interquartile range (25th; 75th percentile) in case of variables showing consistent skewness. Ranges (min to max) were always reported. Differences among groups were accordingly assessed using the parametric ANOVA or the Kruskall-Wallis approach followed by Student *t*-test or the Mann-Whitney U test for pairwise comparisons. Categorical variables were summarized using absolute frequencies and percentages and compared among groups using either the chi-square test or the Fisher exact test when appropriate. Correlations were assessed using the non-parametric Spearman rank correlation coefficient. All tests were two-tailed; a *p*-value of 0.05 was considered significant. No adjustment for multiple comparisons has been undertaken. All statistical analyses were realized with the statistical platform R (The R Formulation for Statistical Computing, Vienna, Austria).

## 3. Results

### 3.1. OSA Is Highly Prevalent in ILDs

Demographics and clinical features of our study population are reported in Table 1. ILD patients were sub-divided in two groups (i.e., IPF and no-IPF). Lung function parameters and main findings of the cardio-respiratory sleep study are shown in Table 2.

Overall, OSA was detected in 79 ILD cases (79%). Of these, 46 patients had a mild disease (58.2%), while 16 (20.3%) and 17 (21.5%) had respectively a moderate and severe OSA (Table 3). Both OSA prevalence and severity were similar in IPF and no-IPF cases. OSA severity distribution in the control group is also reported in Table 3 for comparison. As shown, severe disease was the most prevalent form in the OSA control group as it was recorded in almost the half of cases. In ILD-OSA cases, AHI was positively correlated with age (r = 0.24; *p* = 0.016), while male gender was significantly associated with both AHI (*p* = 0.004) and OSA prevalence (*p* = 0.006). Unlike control OSA patients, only 11 ILD-OSA cases (13.9% vs. 46% in the control group, *p* < 0.005) had a ESS score >10, all of them being affected by moderate-severe OSA.

A condition of NRF was detected in 21 ILD patients and 16 OSA controls. OSA and NRF were associated in 16 ILD patients, with no differences between IPF and no-IPF cases, and in 16 OSA controls (Table 3). Prevalence of NRF was mainly distributed among mild and severe ILD-OSA patients, while it was significantly higher in severe control OSA. Neither OSA/NRF prevalence nor sleep-related indices were correlated with co-morbidities or lung function parameters of ILD-OSA patients.

### 3.2. Description of the Hyperpnea–Hypopnea Periodic Breathing in ILD-OSA

**Figure 1:**
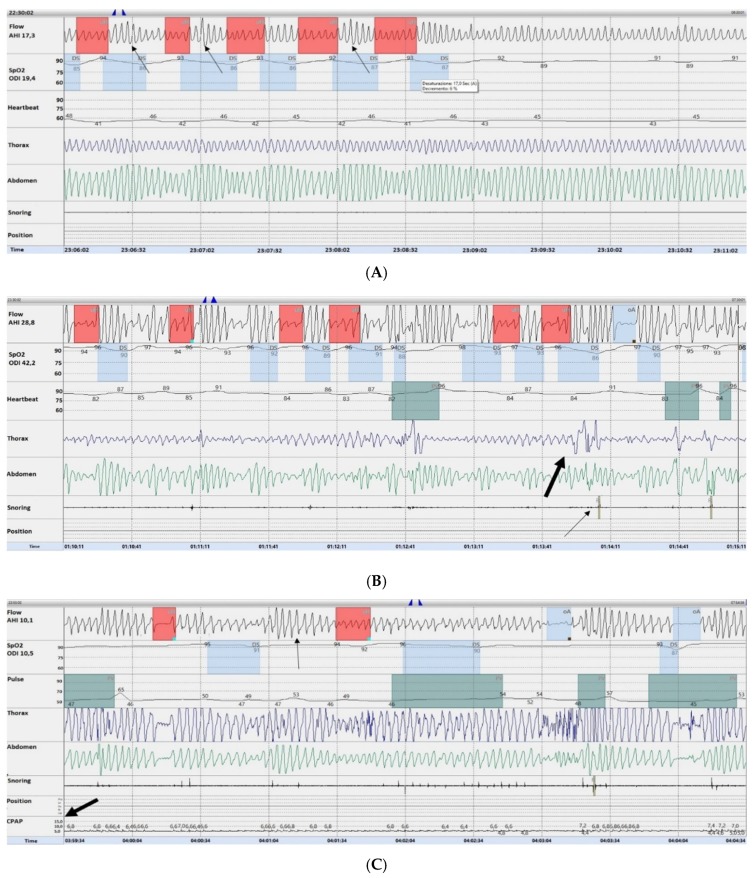
(**A**) Representative 5-min template of the hyperpnea–hypopnea periodic breathing in a patient affected by IPF-OSA. As shown by the red boxes on the flow trace, five hypopneas, each associated with a minimum 4% drop in SpO_2_ (light blue boxes on the SpO_2_ trace) are present. Hypopneas are preceded by hyperpnea events (black arrow). Hypopneas are likely central events as snoring, thoraco-abdominal paradox and increased inspiratory flattening are absent. (**B**) Representative five minutes template of the sleep study in a control OSA patient. Hypopneas (red boxes on the flow trace) are represented by obstructive events as shown by associated snoring (thin arrows), thoraco-abdominal paradox, and increased inspiratory flattening (thick black arrows). (**C**) Representative five minute template of the sleep study in an ILD-OSA patient tested with a continuous positive pressure (CPAP) support. As shown, the hyperpnea–hypopnea periodic breathing (thin black arrow) is still detectable upon CPAP (thick black arrow).

We found that in ILD-OSA patients the respiratory sleep pattern was characterized by a high prevalence of hypopneas over apneas, and that most hypopneas were preceded by a sequence of hyperpnea events with evidence of a sort of periodic pattern. A representative layout of this finding, that we arbitrary defined hyperpnea-hypopnea periodic breathig (HHPB), was recorded in the absence of any paradoxical chest wall and abdomen effort, increased inspiratory flattening and/or snoring, suggesting its likely generation at the central level (Figure 1A). Overall, the length of the HHPB was in all cases found to not exceed 30–32 s. Conversely, only obstructive hypopneas were recorded in pure OSA patients (Figure 1B), as the HHPB was not detected in any case. In addition, recurrence of the HHPB was still appreciable, although to a lesser degree, in ILD-OSA patients while on CPAP, as shown in a representative case in Figure 1C.

### 3.3. Oxygen Resolves the Hyperpnea–Hypopnea Periodic Breathing in ILD-OSA

**Figure 2:**
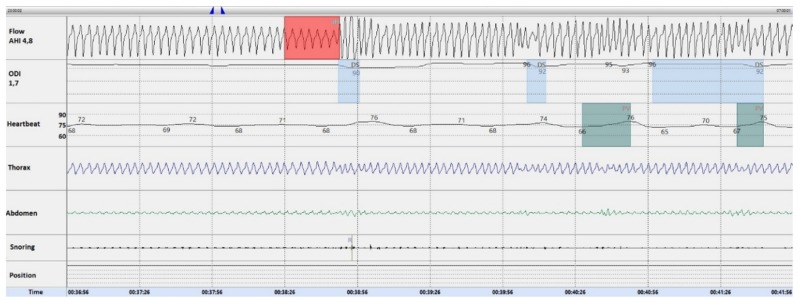
Representative five minutes template of the sleep study in a patient affected by IPF-OSA tested with oxygen supplementation. As shown, the hyperpnea–hypopnea periodic breathing is completely abolished. The red box marks an obstructive hypopnea event.

Khoo et al. reported that transient episodes of hyperpnea can trigger hypopnea events through fluctuations of arterial pCO_2_ [30]. Integrating this evidence with our findings, and assuming that oxygen may correct variations of capnia levels occurring during sleep, home sleep testing was repeated in moderate-severe ILD-OSA with oxygen supplementation. Oxygen titration was performed on an individual basis with a requested mean flow of 2.3 ± 1.4 L/min with nasal cannula. As shown in a representative ILD-OSA patient in Figure 2, oxygen was highly efficacious as allowed the complete disappearance of the HHPB in all tested patients.

Given these observations, we further asked whether the frequency distribution of the HHPB could influence the therapy management of ILD-OSA. Thirty-three ILD patients (42%) with a HHPB associated with an hypopnea/apnea ratio ≥3 had the best response to oxygen with a median residual AHI of 2.6 (1.8–5.6) vs. 28.3 (20.7–37.8) at baseline (*p* < 0.0001). Oxygen was also beneficial in patients with an hypopnea/apnea ratio <3, but the residual AHI was still >5. Patients with a HHPB associated with an hypopnea/apnea ratio ≥ 3 were similarly recorded in the IPF (54.5%) and no-IPF group (45.5%), as shown in Table 4, with most cases being affected by moderate-severe OSA (57.5% vs. 30.5%, *p* = 0.027) (Table 4). Finally, the HHPB associated with an hypopnea/apnea ratio ≥3 was not correlated with any demographic, clinical, or lung function parameter.

## 4. Discussion

To our knowledge, this is the largest prospective study addressing the association of OSA with ILDs, through the inclusion of both IPF and no-IPF patients. It also represents the first effort of describing the frequency distribution and characterization of hypopneas along with treatment implications in this clinical scenario. Despite the strict relationship of sleep disturbances and ILDs was already known in the past [31,32,33,34], awareness of OSA has significantly improved in the last decade with respect to IPF as main co-morbidity [7,8,9,10,11]. Sleep fragmentation, alteration of the sleep respiratory pattern and oxygen saturation decreases are predictors of poor IPF outcome [9,12,14].

We found that OSA was highly prevalent in our patient cohort (79%), while a condition of NRF (alone or in combination with OSA) was detected in a minority of cases (21%). The highest prevalence of OSA (91%) was reported among 31 IPF patients in Greece [12]. Conversely, the lowest prevalence (5.9%) was recorded in a large retrospective US study [35]. In two prospective studies, each including 50 IPF patients, OSA prevalence was of 82.3% and 88%, respectively [6,8]. OSA prevalence in other fibrotic ILDs is poor investigated with estimates not significantly different from IPF [6,13]. In our case series, OSA prevalence had a similar distribution in IPF and no-IPF cases. Unlike previous data [8,10], mild disease was detected in more than half of the patients in both sub-cohorts while frequencies of moderate and severe OSA were slightly higher in IPF (*p* = ns). An estimated prevalence of moderate and severe OSA of respectively 62% and 40% has been recently reported in a French study in 45 IPF cases [36]. Unlike data by Gille et al. [36], OSA severity was not correlated with lung function and co-morbidities in our cohort. Also, OSA was not associated with obesity likely because only a minority of patients (16%) had a BMI greater than 30. In agreement with previous findings, only a minority of patients were symptomatic (13.9%) as assessed by the ESS, and all of them were affected by moderate-severe disease [8,10].

According to previous findings in IPF patients [8,20], we confirm in our cohort that the sleep-related respiratory events in ILD-OSA patients were characterized by a clear predominance of hypopnea events over apneas, with no differences between IPF and no-IPF cases. Notably, the definition of hypopnea is an area of considerable controversy: in our study we used the recommend definition according to the 2007 updated scoring manual [28]. This because the Centers of Medicare and Medical Services currently accept only this definition that is applicable to limited channel home sleep testing [28]. Very interestingly, we also found that most hypopneas were anticipated by hyperpneas configuring a sort of periodic pattern. This finding we arbitrarily defined as ‘hyperpnea–hypopnea periodic breathing’ was likely representing a central event and was completely absent in the control group of pure OSA patients. Also, the HHPB was only partially responsive to CPAP therapy. Altogether these observations were quite intriguing as suggestive of a sort of instability of the sleep breathing pattern in ILD-OSA patients. Certainly, our HHPB seems to mimic the Cheyne-Stokes pattern from which has to be carefully differentiated and suspected in patients with chronic heart failure (CHF). The exclusive presence of hypopneas (and not apneas) and the length of the event not exceeding 30–32 s are the main components that differentiate the HHPB from the Cheyne-Stokes pattern. In our patient cohort, CHF was not an a priori exclusion criterium due to the real-life nature of the study. However, a complete cardiologic evaluation, which is part of the routine diagnostic work-up of our patients, allowed us to exclude this evenience that might have posed some interpretation concerns.

It is likely that hypopneas in ILDs may be facilitated by desaturation events related to ventilation/perfusion abnormalities. Nocturnal hypoxemia is an early event in ILD patients because of a reduced respiratory drive and may account for the generation of hypopneas. However, we suppose that nocturnal hypoxemia may only in part explain our finding. This because only a minority of patients with ILD-OSA had a concomitant condition of nocturnal respiratory failure (<20% in each study sub-group). By the other, the hyperpnea-hypopnea periodic breathing was not recorded in patients with ILDs affected by NRF but not by OSA. Taking into account these considerations, we have thought that a further explanation may be represented by fluctuations of arterial pCO_2_ levels in response to transient hyperpneas. Khoo et al. have reported that the increase in steady-state arterial pCO_2_ accompanying sleep may trigger hyperpneas in systems with reduced chemo-responsiveness in order to reset capnia levels through the induction of compensatory hypopnea events [30]. Our opinion is that ILD-OSA patients may be more susceptible even to little pCO_2_ fluctuations likely because of a high loop gain. Although we did not specifically answer this issue due to technical limitations (no availability of capnography), our belief was, at least in part, explained by the evidence that—despite finding no differences in baseline arterial pCO_2_ levels—median HCO_3_ concentrations were higher in ILD-OSA patients (26 mmol/L (25; 27) vs. 24 (23; 25.5), *p* = 0.04) than in ILD cases without OSA.

In agreement with these considerations, and assuming that oxygen therapy may maintain stable arterial pCO_2_ levels, we found that nocturnal oxygen supplementation completely abolished the HHPB in all tested ILD-OSA patients. The best effect was achieved in patients with a HHPB associated with an hypopnea/apnea ratio ≥3, as they had a median residual AHI <5 upon oxygen supplementation. Such a threshold was not arbitrarily set up, but arose from direct clinical observation. It is simply indicative of the fact that, at least in our cohort, oxygen responsiveness in terms of AHI reduction/normalization was related to the prevalence of hypopneas over apneas at baseline. The identification of a reference threshold in larger patient populations may be of clinical relevance in the future for differentiating ILD-OSA patients with the HHPB who may benefict from oxygen therapy. It is worth noting that oxygen resolved not only desaturation events but also flow limitation, which is the main component of the hypopnea definition (even when associated to arousals instead of desaturation). This means that, despite home sleep testing does not allow the detection of arousals, it is very unlikely that any (unrecorded) residual arousal was related to respiratory events in our setting. Altogether these data are of utmost clinical relevance as correction of intermittent desaturation events may be crucial for preventing potentially severe complications like pulmonary hypertension [12], not excluding the beneficial effect of oxygen on the ventilation/perfusion mismatch. In addition, as oxygen is better tolerated than CPAP, we believe that differentiating patients with the HHPB also raises the possibility of increasing the rate of treatment compliance in selected ILD-OSA patients. Supplemental oxygen is currently used as salvage treatment in OSA patients not tolerant of CPAP [37,38,39]. Recently, Sands et al. have reported that in OSA patients an elevated loop gain along with other traits including better pharynx collapsibility and compensation are predictive of oxygen responsiveness [40]. Confirmation studies are encouraged to definitely address this potential treatment option. Similarly, since this was not the aim of our study, the beneficial effect of oxygen on the control of the OSA related daily sympoms has to be investigated in longitudinal cohorts.

Lastly, a further limitation of our study includes the single center setting and the heterogeneity of no-IPF patients. The last one may also be considered as an advantage over previous studies exclusively restricted to IPF. Also, as we did not perform polysomnography we cannot directly address the question whether the HHPB was associated with sleep stages. However, taking into account a previous study by Lancaster et al. [8], it seems that there is no association between the distribution of hypopnea events and the different stages of sleep in IPF patients. In our cohort, the occurrence of such an association is also high unlikely as the HHPB was widely distributed during the whole sleep registration time. Certainly, polysomnography studies will definitely address this issue.

## 5. Conclusions

In conclusion, the generating pathway of the HHPB should be carefully investigated in ILD-OSA. Our feeling is that this finding may be typical of ILD-OSA. At the same time, however, we believe that ad hoc studies addressing whether this is also present in different OSA patient populations with associated lung disorders other that ILDs have to be encouraged. Certainly, our report demonstrates that home sleep polygraphy, which represents the first-line diagnostic tool for OSA most widely used in clinical practice, is extremely useful as allows clinicians to easily differentiate ILD-OSA patients with the HHPB. Future validation efforts are needed as this finding may have significant implications in terms of real-life therapy management and adherence outcomes.

## Figures and Tables

**Table 1:** Demographics and clinical features of the study population.

Variables	Controls (n = 50)	IPF (n = 54)	No-IPF (n = 46)	Overall *p*
Gender, female (n)	17 (34)	14 (25.9)	17 (37)	0.54
Age (years)	66.5 ± 7.9	69.9 ± 7.1	66.8 ± 8.8	0.52
Smoking habit (n)				**<0.001**
No smokers	19 (38)	15 (27.8)	17 (37.8)	
Former smokers	22 (44)	39 (72.2)	19 (42.2)	
Smokers	9 (18)	0 (0)	9 (20)	
Pack/yr	25 (0; 30)	36 (20; 60)	30 (11.5; 37.5)	0.315
BMI (Kg/m^2^)	31 (26.5; 35)	28.7 (25.1; 30.7)	28 (25.7; 31.1)	**0.034**
Co-morbidities (n)				
Systemic arterial hypertension	29 (58)	32 (59.3)	26 (56.5)	0.978
Gastro-esophageal reflux	6 (12)	24 (44.4)	14 (30.4)	**0.001**
Type II diabetes	9 (18)	14 (25.9)	4 (8.7)	0.082
Cardiovascular diseases (CVD) *	10 (20)	13 (24.1)	10 (21.7)	0.883
Thyroid disease	4 (8)	3 (5.68)	3 (6.5)	0.92
Pulmonary hypertension	0 (0)	5 (9.3)	3 (6.5)	0.076
Epworth sleepiness scale score	10 (7.8; 12)	6 (4; 8)	6 (4; 8)	**<0.001**

Data are expressed as absolute number (%), mean ± SD, median (IQR 25; IQR75), where appropriate. * CVD included previous ischemic heart disease, mild-to moderate valvulopathies and atrial arritmias (mainly atrial fibrillation). Statistically significant results (*p* < 0.05) are reported in bold. Abbreviations: n = number; BMI = body mass index.

**Table 2:** Lung function and sleep-related events in the study population.

Parameter	Controls (n = 50)	IPF (n = 54)	No-IPF (n = 46)	Overall *p*
Arterial pO_2_ (mmHg), at rest at 21% FiO_2_	69.7 ± 12	70.2 ± 10.8	69.1 ± 13.3	**<0.001**
SpO_2_ (%), at rest at 21% FiO_2_	96 (94; 97)	96 (93.6; 97.4)	96 (93.8; 97)	**<0.001**
FVC (% pred)	69.6 ± 22.1	71.5 ± 23.2	67.4 ± 20.8	**<0.001**
TLC (% pred)	60.5 ± 19	60 ± 19.2	61.3 ± 19	**0.003**
RV (% pred)	49.5 (37; 60.5)	48 (30.5; 58.5)	50 (40; 68)	**<0.001**
DLCO_sb_ (% pred)	48.6 ± 19	47.5 ± 19.4	50 ± 18.7	**<0.001**
6-MWT distance (meters)	410 (318; 528)	443 (308; 535)	401 (346; 507)	**0.01**
TST (minutes)	390 (325; 426)	438 (391; 466)	446 (402; 458)	**<0.001**
Supine time (%)	52 (21; 81)	12 (3; 26)	46 (19; 65)	**0.001**
AHI (events/hour)	28.6 (20.6; 68.3)	10.8 (5.3; 26.9)	10.8 (7.4; 15.8)	**<0.001**
OAI (events/hour)	23.9 (15.5; 65.8)	2.7 (0.98; 6.7)	3 (1; 7.8)	**<0.001**
CAI (events/hour)	0.1 (0; 0.8)	0.05 (0; 0.4)	0 (0; 0.1)	0.066
Supine AHI (events/hour)	37.6 (12; 60)	17 (4.8; 30.8)	13.5 (2.7; 27.5)	**0.012**
HI (events/hour)	7.7 (3.7; 13.1)	7.2 (3.3; 11.5)	7 (3.2; 12.4)	0.892
ODI (events/hour)	33.3 (14; 66.5)	15.9 (7.5; 29)	12 (7.4; 19.1)	**<0.001**
t90 (%)	15.5 (2.8; 37.9)	6 (1; 26.2)	4.5 (0; 20.5)	0.139
Nadir SpO_2_ (%)	72.5 (60.2; 79)	77.5 (67; 82)	77.5 (70; 82.2)	0.055

Data are expressed as mean ± SD or as median (IQR25; IQR75) where appropriate. Statistically significant results (*p* < 0.05) are reported in bold. Abbreviations: pO_2_ = oxygen partial pressure; FiO_2_ = fraction of inhaled oxygen; SpO_2_ = arterial oxygen saturation; FVC = forced vital capacity; TLC = total lung capacity; RV = residual volume; DLCO_sb_ = single breath diffusion lung capacity for carbon monoxide; 6-MWT = six minute walking test; TST = total sleep time; AHI = apnea/hypopnea index; OAI = obstructive apnea index; CAI = central apnea index; HI = hypopnea index; ODI = oxygen desaturation index; t90% = percentage of time spent with SpO_2_ < 90%; SpO_2_ = arterial oxygen saturation.

**Table 3:** Prevalence of OSA in the study population

Parameter	Controls	IPF	No-IPF	Overall *p*
OSA	50 (100)	42 (77.8)	37 (80.4)	**<0.001**
OSA severity				**<0.001**
Mild	8 (16)	21 (50)	25 (67.6)	
Moderate	18 (36)	10 (23.8)	6 (16.2)	
Severe	24 (48)	11 (26.2)	6 (16.2)	
NRF	16 (32)	12 (22.2)	9 (19.6)	0.319
OSA + NRF	32 (21.3)	10 (18.5)	6 (13)	0.069
OSA severity + NRF				**0.01**
Mild	0 (0)	4 (40)	3 (50)	
Moderate	2 (12.5)	1 (10)	1 (16.7)	
Severe	14 (87.5)	5 (50)	2 (33.3)	

Data are expressed as absolute number (%). Statistically significant results (*p* < 0.05) are reported in bold. Abbreviations: SRDs = sleep related disorders; OSA = obstructive sleep apnea; NRF = nocturnal respiratory failure.

**Table 4:** Distribution of the hyperpnea–hypopnea periodic breathing in ILD-OSA patients according to the hypopnea/apnea ratio.

Parameter	HHPB (with an Hypopnea/Apnea Ratio < 3)	HHPB (with an Hypopnea/Apnea Ratio ≥ 3)	*p*
Diagnosis			1
IPF	25 (53.7)	18 (54.5)	
No-IPF	21 (46.3)	15 (45.5)	
OSA severity			**0.027**
Mild	32 (69.6)	14 (42.4)	
Moderate	5 (10.9)	11 (33.3)	
Severe	9 (19.6)	8 (24.2)	

Data are expressed as absolute number (%). Statistically significant results (*p* < 0.05) are reported in bold. Abbreviations: ILD = interstitial lung disease; OSA = obstructive sleep apnea; HHPB = hyperpnea–hypopnea periodic breathing; IPF = idiopathic pulmonary fibrosis.
